# Brugia Rapid™ antibody responses in communities of Indonesia in relation to the results of ‘transmission assessment surveys’ (TAS) for the lymphatic filariasis elimination program

**DOI:** 10.1186/s13071-015-1093-x

**Published:** 2015-10-01

**Authors:** Rita M. Dewi, Sekar Tuti, Sitti Ganefa, Chairiyah Anwar, Ria Larasati, Endah Ariyanti, Herty Herjati, Molly Brady

**Affiliations:** National Institute of Health Research and Development, Indonesia Ministry of Health, Jl. Percetakan Negara No. 29, Jakarta, 10560 Indonesia; Sub-Directorate of Filariasis & Helminthiasis Control, Directorate of Vector Borne Disease Control, Directorate General of Communicable Disease and Environmental Health, Indonesia Ministry of Health, Jl. Percetakan Negara No. 29, Jakarta, 10560 Indonesia; RTI International-Indonesia, Menara Thamrin, 15th Floor, Suite 1503, Jl. M.H. Thamrin Kav. 3, Jakarta, 10250 Indonesia; RTI International, 701 13th Street NW, Suite 750, Washington, DC 20008 USA

**Keywords:** Lymphatic filariasis, Elimination, Diagnostic tests, Program evaluation

## Abstract

**Background:**

The Global Programme to Eliminate Lymphatic Filariasis recommends the transmission assessment survey (TAS) as the preferred methodology for determining whether mass drug administration can be stopped in an endemic area. Because of the limited experience available globally with the use of Brugia Rapid™ tests in conducting TAS in *Brugia spp.* areas, we explored the relationship between the antibody test results and *Brugia spp.* infection as detected by microfilaremia in different epidemiological settings.

**Methods:**

The study analyzes the Brugia Rapid™ antibody responses and microfilaremia in all ages at three study sites in: i) a district which was classified as non-endemic, ii) a district which passed TAS, and iii) a district which failed TAS. Convenience sampling was done in each site, in one to three purposefully selected villages with a goal of 500 samples in each district.

**Results:**

A total of 1543 samples were collected from residents in all three study sites. In the site which was classified as non-endemic and where MDA had not been conducted, 5 % of study participants were antibody positive, none was positive for microfilaremia, and age-specific antibody prevalence peaked at almost 8 % in the 25–34 year-old age range, with no antibody-positive results found in children under eight years of age. In the site that had passed TAS, 1 % of participants were antibody positive and none was positive for microfilaremia. In the site which failed TAS, 15 % of participants were antibody positive, 0.2 % were microfilaremic, and age-specific antibody prevalence was highest in 6–7 year olds (30 %), but above 8 % in all age levels above 8 years old.

**Conclusions:**

These results from districts which followed the current WHO guidance for mapping, MDA, and implementing TAS, while providing antibody profiles of treated and untreated populations under programmatic settings, support the choice of antibody prevalence in the 6- and 7-year-old age group in TAS for making stopping MDA decisions. Since only one study participant was microfilaremic, no conclusions could be drawn about the relationship between antibodies and microfilaremia and further longitudinal studies are required to understand this relationship.

## Background

### Lymphatic filariasis

Lymphatic filariasis (LF), also known as elephantiasis, is a parasitic disease caused by three species of parasitic worms – *Wuchereria bancrofti*, *Brugia malayi*, and *Brugia timori* – and transmitted by mosquitoes. It can cause clinical complications of lymphedema and hydrocele, making it one of the most disabling diseases in the world. LF is endemic in 73 countries, with 57 % of the at-risk population living in the World Health Organization (WHO)’s Southx-East Asia Region, which includes three of the largest endemic countries - India, Bangladesh and Indonesia [1].

In 1997, the World Health Assembly passed resolution 50.29, calling for the elimination of LF as a public-health problem [2]. The strategy for interrupting transmission of LF includes the sequential activities of mapping, mass drug administration (MDA), post-MDA surveillance and validation. In 2011, WHO published updated monitoring and evaluation guidance, based on operational research conducted mostly in *W. bancrofti* areas [3, 4]. This guidance introduced the transmission assessment survey (TAS), an impact survey designed to determine whether prevalence has been lowered to such a level that MDA could be stopped and recrudescence would not occur. This infection level is likely to be reached after five to six annual MDA rounds with effective coverage of at least 65 % of the total population. The TAS uses a population-based cluster-sampling methodology to estimate prevalence among 6–7 year old children in an evaluation unit.

Evaluation units ‘pass’ TAS if the number of positive children are equal to or below a defined threshold (the ‘critical cut off’), determined by the size of the population and powered so that the evaluation unit has at least a 75 % chance of passing if the true prevalence is half the critical threshold level (defined as 2 % in areas where *Culex*, *Anopheles*, and *Mansonia* are the mosquito species serving as the vectors of the infection) [3].

In *W. bancrofti* areas, prevalence is measured using antigen, detected using a point-of-care test, the immunochromatographic test. An antigen detection test is preferred for measuring prevalence during TAS as it is more sensitive to detection than microfilaremia and can be measured using a rapid test at any time of day, while measurement of microfilaremia requires laboratory tests, usually with blood collected in late evenings [5].

For *Brugia spp.* infections, however, there is no antigen-detection test, so WHO recommends the use of the Brugia Rapid™ *antibody*-detection test [Reszon Diagnostics International, Subang Java, Selangor, Malaysia] in *Brugia spp.* areas. The Brugia Rapid™ test uses a recombinant *B. malayi* antigen *Bm*R1 and has been previously evaluated for sensitivity and specificity [6, 7]. Antibody testing is useful in evaluating programmatic endpoints as antibodies are more sensitive than microfilaremia or antigenaemia and develop earlier in the course of the infection [8–10].

Although “critical cut off” thresholds identical to those in *W. bancrofti* areas are used, experience with these thresholds for *antibody* prevalence in the *Brugia spp.* areas is, unfortunately, very limited. WHO TAS guidance, while recognizing that these thresholds might be conservative, as antibody levels are most likely to be higher than antigen levels, suggested operational research to better understand the “precise relationship between antibody prevalence in children and sustainability of transmission” [3]. The present study, conducted under programmatic conditions, profiles a descriptive analysis of the Brugia Rapid™ antibody responses and microfilaremia in both children and adults in communities being assessed for their non-endemic or post-MDA LF status to better understand this relationship.

### Indonesia context

Indonesia has one of the heaviest burdens of LF globally, spread throughout 241 districts, with 86 million people at risk. LF in Indonesia is caused by all three parasites and transmitted by a variety of mosquito vectors. MDA using diethylcarbamazine citrate (DEC) (6 mg/kg) and albendazole (400 mg) has been funded and implemented by district governments, with supervision from provincial health offices or the central LF team. The national LF program’s strategy recommends annual population registration by kaders (community health volunteers), directly-observed treatment at MDA posts by health staff and kaders, and door-to-door sweeping at the conclusion of the campaign. In 2012, TAS were implemented following new WHO guidance in 14 districts throughout Indonesia to determine whether or not prevalence had fallen to levels such that MDA could be confidently stopped.

## Methods

Because of the limited experience available globally with the use of Brugia Rapid™ tests in conducting TAS in *Brugia spp.* areas it was especially important to the Indonesia LF program to explore the relationship between the antibody test results and *Brugia spp.* infection as detected by microfilaremia. Therefore, such comparisons were explored in three study sites: i) in all ages in a district which was classified as non-endemic, ii) in all ages in a district which passed TAS, and iii) in all ages in a district which failed TAS.

### Study area

One to three communities were purposively selected in each study site. All study sites had records of *B. malayi* found in blood slides in earlier studies, with no record of people found with *W. bancrofti*. MDA was started in study sites classified as endemic, i.e., those with baseline microfilaremia prevalence ≥1 %. The study site that had been classified as non-endemic and where MDA had not been conducted, Payakumbuh district in Sumatra Barat province, had a baseline microfilaremia prevalence of 0.0 % (0/600) in 2012. In this study site, one community was selected after discussion with the district health office based on highest potential risk of LF transmission. Without known clinical cases in the district, the community was chosen because of its proximity to and migration between villages with chronic cases in a neighboring LF-endemic district.

The second site, Enrekang district in Sulawesi Selatan province, with a baseline microfilaremia prevalence of 1.06 % (4/377) in 2006, passed TAS after five rounds of effective MDA. MDA rounds were implemented between 2007 and 2011, with the reported coverage of the total population ranging from 70 to 100 %. In 2011, sentinel and spot-check sites found 0 % microfilaremia in 500 and 400 samples, respectively. During TAS in 2012, only one antibody-positive result was found from a total of 1540 first- and second-grade children. Therefore, the TAS passed and Enrekang stopped MDA. In Enrekang, two communities were selected for this study – one with the antibody-positive student from the 2012 TAS and one that had positive microfilaremia results from a previous survey.

The district which failed TAS, Pasaman Barat in Sumatra Barat province, had a baseline microfilaremia prevalence of 18.5 % (61/330) in one site in 2002. MDA was implemented on a sub-district basis from 2004 to 2010, with reported coverage rates ranging from 47 to 95 % of the total district population and all sub-districts reporting taking part in five rounds. TAS was undertaken in 2012 after assessments in 2010 in sentinel and spot-check sites found 0.0 % (0/539) and 0.5 % (2/383) microfilaremia, respectively. During TAS, 64 antibody-positive results were found from a total of 1353 first- and second-grade children. The three selected communities in Pasaman Barat were those villages with antibody-positive students from the school with the most positive Brugia Rapid™ test results from the 2012 TAS.

### Sample collection

Two-person research teams from the National Institutes of Health Research and Development (NIHRD) in Jakarta, the section of the Ministry of Health of Indonesia responsible for implementing health research, accompanied Ministry of Health national LF elimination program staff to the communities to collect samples in September 2013. Convenience sampling was done in each community, with a goal of 500 samples in each community from residents ≥5 years of age. Antibody and microfilaremia testing was done on all participants. Data – including name, age, sex, and address – was collected on paper forms. The village head and health volunteers announced the survey to the community before the research team arrived and every individual who agreed to participate was tested.

### Blood tests

For microfilaremia testing, 60 microliters of finger prick blood was taken between the hours of 10 pm and 2 am, drawn into three lines on a slide, and stained with Giemsa in the field after 24 h. NIHRD staff in the field and in Jakarta read the slides. Ten percent of negative slides and all positive results were cross-checked by the lead NIHRD team member. Health center staff were informed of the microfilaremia positive results so they could follow up with treatment with albendazole and DEC. Blood was taken for antibody testing at the same time as blood was taken for microfilaremia testing. Antibody positivity was assessed by the Brugia Rapid™ cassette test, following test instructions, scoring positive, negative or ‘invalid’ due to lack of positive control line on the test, blood not flowing up or clearing from the strip, or insufficient blood applied to the test. Thirty-five microliters of whole blood from finger prick was tested, with tests read visually at 25 min.

### Analysis

Data was entered into Excel by NIHRD staff. Analysis was done in Excel to determine breakdown of age, gender, antibody-positivity and microfilaremia in each district. Age-specific antibody prevalence also was calculated for each study site.

### Ethics statement

Ethical approval was given by the Indonesia Institutional Review Board. Informed consent was given in community surveys by the head of village, as is typical practice by NIHRD.

## Results

A total of 1543 samples were collected from residents in all three study sites. The median age in the study sites ranged from 21 years to 33 years. Overall demographic results from the study are presented in Table 1.

**Table 1:** Demographics of study participants, by study site

District	Category	Sample size	Median age (years)	Number (%) of participants by age range (in years)								Number (%) of participants by sex	
				≤5	6–7	8–14	15–24	25–34	35–44	45–54	≥55	Female	Male
Payakumbuh	Non-endemic	506	33	3 (0.6)	21 (4.2)	96 (19.0)	74 (14.6)	76 (15.0)	92 (18.2)	80 (15.8)	64 (12.6)	261 (51.6)	245 (48.4)
Enrekang	Pass TAS	514	27	16 (3.1)	37 (7.2)	139 (27.1)	53 (10.3)	53 (10.3)	80 (15.6)	70 (13.6)	65 (12.7)	232 (45.1)	282 (54.9)
Pasaman Barat	Fail TAS	523	21	3 (0.6)	33 (6.3)	169 (32.3)	70 (13.4)	73 (14.0)	93 (17.8)	58 (11.1)	24 (4.6)	335 (64.3)	186 (35.7)

### Non-endemic site

**Fig. 1:**
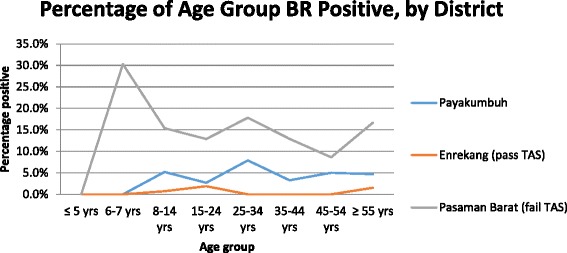
Age-specific prevalence for LF antibody, by study site

In Payakumbuh, a total of 23 (5 %) study participants were antibody positive; however, none was positive for microfilaremia. Almost 6 % of females and 3 % of males were antibody positive (Table 2). Age-specific antibody prevalence peaked at almost 8 % in the 25–34 year-old age range, with no antibody-positive results found in children under eight years of age (Fig. 1).

**Table 2:** LF antibody positivity, by site and age group

District	Category	Number (%) of antibody-positive participants	Number (%) of antibody-positive participants, by age range (in years)								Number (%) of antibody-positive participants, by sex	
			≤5	6–7	8–14	15–24	25–34	35–44	45–54	≥55	Female	Male
Payakumbuh	Non-endemic	23 (5)	0 (0)	0 (0)	5 (5.2)	2 (2.7)	6 (7.9)	3 (3.3)	4 (5.0)	3 (4.7)	15 (5.7)	8 (3.3)
Enrekang	Pass TAS	3 (1)	0 (0)	0 (0)	1 (0.7)	1 (1.9)	0 (0)	0 (0)	0 (0)	1 (1.5)	2 (0.9)	1 (0.4)
Pasaman Barat	Fail TAS	79 (15)	0 (0)	10 (30.3)	26 (15.4)	9 (12.9)	13 (17.8)	12 (12.9)	5 (8.6)	4 (16.7)	47 (14.0)	32 (17.2)

### Post-MDA, passing TAS site

Results from Enrekang district showed three (1 %) participants were antibody positive, 21 (4 %) had invalid Brugia Rapid™ tests, and 490 (95 %) were antibody negative. Invalid tests were not repeated due to lack of extra Brugia Rapid™ tests. All 514 participants were negative for microfilaremia. The results are shown in Fig. 1.

### Post-MDA, failing TAS site

In our study, 79 (15 %) of participants in Pasaman Barat were antibody positive, one (0.2 %) had an invalid Brugia Rapid™ test, and 443 (85 %) were antibody negative. Overall almost 65 % of participants were female; however, 33 % of 6- and 7-year-olds and 51 % of 8- to 14-year-olds were female. One participant, a 22-year-old male, was positive for *B. malayi* microfilaremia (as well as antibodies). Age-specific antibody prevalence was highest in 6–7 year olds (30 %), although this was a small sample, with only 33 (6.3 %) of total participants included in this age range. Prevalence was above 8 % in all age levels above 8 years old (Fig. 1).

## Discussion

A better understanding of antibody responses in all ages is critical for determining the appropriateness of current WHO guidelines in *Brugia spp.* areas. Our study found antibody results in all ages consistent with the outcomes of the previous mapping and TAS activities. The district which failed TAS (Pasaman Barat) had the highest antibody prevalence of the three study sites. In contrast, in the district which passed TAS (Enrekang), only a few antibody-positive individuals were identified. While a higher number of invalid tests were reported in Enrekang, two of these were in the 6- and 7-year-old age group. Even if these children actually were antibody positive, it would still be markedly lower in Enrekang (5 %) than in Pasaman Barat (30 %).

While antibody-positive individuals were found in the district originally classified as non-endemic based on microfilaremia prevalence (Payakumbuh), overall antibody prevalence in this study site was low. The overall antibody prevalence in Enrekang (the district that passed TAS) was the lowest among all the three districts studied. These low levels are the consequence of successful implementation of effective MDA that reduced exposure to the parasite. The next highest levels seen in Payakumbuh (the non-endemic district), reflect low level exposure to the parasite (possibly from neighboring endemic districts) since no interventions were undertaken in this area. The Ministry of Health LF elimination program’s surveillance activities require monitoring of non-endemic districts, which will help to identify if interventions are needed in the future.

The TAS guidance recommends using a survey population of 6- and 7-year-olds because “they should be protected from LF infection if the MDAs have been successful in interrupting transmission. Antigenemia in young children is a marker for relatively recent events of transmission, while antigenemia in older children or adults may be related to infections that occurred before MDA” [3]. How applicable the 6- and 7-year-old age group is when antibody diagnostics are used is still a matter of some debate. A multicenter evaluation of diagnostic tools in *W. bancrofti* areas after five years of MDA found antibody reactivity to the recombinant antigen Bm14 to be similar among all age groups: 30 % (0–5 years), 32 % (6–10 years), and 37 % (all ages) [5]. Similarly, research in the Cook Islands found that Wb123 antibody prevalence in children did reflect *W. bancrofti* infection and transmission in the overall population [11]. Not surprisingly, antibody prevalence before treatment in Papua New Guinea, measured by IgG4 antibodies to Bm14 detected by ELISA, was higher in adults (59.3 %) than in children 6- to 10-years-old (35.8 %). In addition, after three rounds of MDA, antibody prevalence decreased faster in children less than 11 years of age than in the total population; perhaps due to lower infection intensity or shorter duration of infection in the children than in adults [12].

Previous studies which examined antibody prevalence in *Brugia spp.* areas found that antibody levels in children (defined as under 15 or under 10 years old) were similar to levels in adults in pretreatment situations [6, 13, 14]. However, there is limited information on the age-specific prevalence of antibody in post-treatment *Brugia spp.* areas – in particular, in areas that had conducted mass drug administration and not individual treatment. Supali, et al., using a dipstick version of the Brugia Rapid™ test, showed a decrease in antibody prevalence from 80 % pretreatment to 6.4 % post-MDA in Alor Island, Indonesia after six rounds of MDA [15]. A study in Sarawak, Malaysia that used Brugia Rapid™ cassette tests found baseline rates of 70 % antibody prevalence among all ages, which fell to 3.5 % after three years of treatment [7]. The authors reported a non-significant difference between post-MDA antibody levels in individuals less than 21 years of age (4.9 %) and in adults (8.7 %).

The relationship between antibody positivity and microfilaremia has been examined in only a few studies so far. While antibody tests detected 10 times and 50 % more antibody-positive individuals than microfilaremic individuals (by thick smear examination) in studies reported by Jamail [14] and Noordin [7] respectively, the antibody to microfilaremia ratio was 3:1 in a study by Supali [15]. The relationship between antibody positivity and microfilaremia could not be analyzed in our study because only one microfilaremic individual was found in all three study sites. The low level of microfilaremia in all the three sites suggest that infections are still light (even in Pasaman Barat) since antibody is known to be detected earlier and with greater sensitivity than antigenemia or microfilaremia [13].

## Conclusions

The results of our study have important messages for MDA programs in *Brugia spp.* areas, particularly because the study was conducted under programmatic conditions according to WHO guidelines for mapping and stopping MDA. In the TAS-failing Pasaman Barat district, although fewer individuals were tested in the 6- and 7-year-old age group, 30.3 % of them were antibody positive, a level even higher than those in all other age groups (8.6 % to 17.8 %). The antibody findings in the older age groups are consistent with infection having occurred in the past, while the results in 6- and 7-year-olds (born after the start of MDA in 2004) agree with the TAS result suggesting that transmission is still ongoing and that MDA should be continued. Conversely, in Enrekang and Payakumbuh, the results in all ages appear to indicate that these areas have minimal, if any, ongoing transmission and do not need MDA. Notably, this conclusion could be drawn from the results in 6- and 7-year-olds *only* in these two study sites. Taken together, these observations strongly support the current WHO recommended strategy of using antibody levels in the 6-and 7-year-old age group for stopping MDA based on TAS.

Clearly, further follow up is required not only to assess the antibody profile in these communities but also the effect of interventions in the long term. Longitudinal collection of data planned by the Indonesian national LF elimination program includes follow up of antibody levels in Pasaman Barat after two more rounds of MDA. Similarly, it would be important to include Payakumbuh in ongoing LF surveillance since the antibody-positivity levels are suggestive of transmission in the past and migration to Payakumbuh from known endemic districts is known to exist. Such a strategy will help in early identification of infection of levels that require institution of MDA in the district.

While the results of our study provide epidemiological support for the use of antibody levels to determine “critical cut-off thresholds” in *Brugia spp.* areas, further comprehensive studies using population-based surveys will undoubtedly contribute significantly to our growing understanding of antibody positivity and its practical usefulness at all stages of the LF program.

## Abbreviations

DEC: Diethylcarbamazine citrate
LF: Lymphatic filariasis
MDA: Mass drug administration
NIHRD: National Institute for Health Research and Development
TAS: Transmission assessment survey
WHO: World Health Organization
